# Impaired Functional T-Cell Response to SARS-CoV-2 After Two Doses of BNT162b2 mRNA Vaccine in Older People

**DOI:** 10.3389/fimmu.2021.778679

**Published:** 2021-11-16

**Authors:** Julie Demaret, Bénédicte Corroyer-Simovic, Enagnon Kazali Alidjinou, Anne Goffard, Jacques Trauet, Sophie Miczek, Fanny Vuotto, Arnaud Dendooven, Dominique Huvent-Grelle, Juliette Podvin, Daniel Dreuil, Karine Faure, Dominique Deplanque, Laurence Bocket, Alain Duhamel, Julien Labreuche, Annie Sobaszek, Michael Hisbergues, Francois Puisieux, Myriam Labalette, Guillaume Lefèvre

**Affiliations:** ^1^ Institut d’Immunologie, U1286 - INFINITE - Institute for Translational Research in Inflammation Inserm Univ. Lille, Centre Hospitalier Universitaire (CHU) Lille, Lille, France; ^2^ Pôle de Gériatrie, Hôpital Gériatrique Les Bateliers, Centre Hospitalier Universitaire (CHU) de Lille, Université de Lille, Lille, France; ^3^ Faculté de Médecine, Laboratoire de Virologie ULR3610, Univ Lille, Centre Hospitalier Universitaire (CHU) Lille, Lille, France; ^4^ Université Lille, Centre Nationale de la Recherche Scientifique (CNRS), Inserm, Centre Hospitalier Universitaire (CHU) Lille, Institut Pasteur de Lille, U1019 - Unité Mixte de Recherche (UMR) 8204 - Centre d’Infection et d'Immunité de Lille (CIIL)-Centre d’Infection et d’Immunité de Lille, Lille, France; ^5^ Médecine et santé-travail, Univ. Lille, Centre Hospitalier Universitaire (CHU) Lille, ULR 4483, IMPact de l’Environnement Chimique sur la Santé (IMPECS), Lille, France; ^6^ Département de Maladies Infectieuses, Centre Hospitalier Universitaire (CHU) Lille, Lille, France; ^7^ Centre d’Investigation Clinique (CIC) 1403 - Clinical Investigation Center, Univ. Lille, Inserm, Centre Hospitalier Universitaire (CHU) Lille, Lille, France; ^8^ EA 2694 - Santé publique: épidémiologie et qualité des soins, Université de Lille, Centre Hospitalier Universitaire (CHU) Lille, Lille, France; ^9^ Centre de Ressources Biologiques, Université Lille, Centre Hospitalier Universitaire (CHU) Lille, Lille, France

**Keywords:** SARS – CoV – 2, vaccine, older people and ageing, T cells response, mRNA vaccination

## Abstract

Long-term care facility (LTCF) older residents display physiological alterations of cellular and humoral immunity that affect vaccine responses. Preliminary reports suggested a low early postvaccination antibody response against severe acute respiratory syndrome coronavirus 2 (SARS-CoV-2). The aim of this study was to focus on the specific T-cell response. We quantified S1-specific IgG, neutralizing antibody titers, total specific IFNγ-secreting T cells by ELISpot, and functionality of CD4^+^- and CD8^+^-specific T cells by flow cytometry, after two doses of the BNT162b2 vaccine in younger and older people, with and without previous COVID-19 infection (hereafter referred to as COVID-19-recovered and COVID-19-naive subjects, respectively). Frailty, nutritional, and immunosenescence parameters were collected at baseline in COVID-19-naive older people. We analyzed the immune response in 129 young adults (median age 44.0 years) and 105 older residents living in a LCTF (median age 86.5 years), 3 months after the first injection. Humoral and cellular memory responses were dramatically impaired in the COVID-19-naive older (*n* = 54) compared with the COVID-19-naive younger adults (*n* = 121). Notably, older participants’ neutralizing antibodies were 10 times lower than the younger’s antibody titers (*p* < 0.0001) and LCTF residents also had an impaired functional T-cell response: the frequencies of IFNγ^+^ and IFNγ^+^IL-2^+^TNFα^+^ cells among specific CD4^+^ T cells, and the frequency of specific CD8^+^ T cells were lower in COVID-19-naive older participants than in COVID-19-naive young adults (*p* < 0.0001 and *p* = 0.0018, respectively). However, COVID-19-recovered older participants (*n* = 51) had greater antibody and T-cell responses, including IFNγ^+^ and IFNγ^+^IL-2^+^TNFα^+^-specific CD4^+^ T cells (*p* < 0.0001), as well as TNFα^+^-specific CD8^+^ T cells (*p* < 0.001), than COVID-19-naive older adults. We also observed that “inflammageing” and particularly high plasma levels of TNFα was associated to poor antibody response in the older participants. In conclusion, our results show that the COVID-19-naive older people had low counts and impaired specific CD4^+^ and CD8^+^ T cells, in addition to impaired antibody response, and that specific studies are warranted to assess the efficiency of SARS-CoV-2 mRNA-based vaccines, as in other immunocompromised subjects. Our study also shows that, despite their physiological alterations of immunity, vaccination is highly efficient in boosting the prior natural memory response in COVID-19-recovered older people.

## Introduction

Since the emergence of severe acute respiratory syndrome coronavirus 2 (SARS-CoV-2) and the beginning of the worldwide coronavirus disease 2019 (COVID-19) pandemic, unprecedented efforts have been made to develop vaccines. Considered among the most at risk of developing severe COVID-19, long-term care facility (LTCF) older residents were among the first to be vaccinated. In addition to age, older adults usually cumulate other risk factors for COVID-19 and death, including diabetes, hypertension, cardiovascular disease, and/or malignancy (1). Furthermore, the closed environment and the relative inability of residents to adopt preventive health measures led to numerous outbreaks in LTCFs worldwide (1). For these reasons, there were high hopes for anti-SARS-CoV-2 vaccines, especially among the older and healthcare workers (HCW) in LTCFs. However, the older display physiological alterations of cellular and humoral immunity that affect vaccine responses (2, 3), and, due to their age and frailty, they were not included in clinical trials evaluating the BNT162b2 mRNA vaccine (4–7).

The aims of this study were (i) to assess the specific memory humoral and cellular response after two doses of the BNT162b2 mRNA vaccine in older LTCF residents in comparison with HCWs, with a focus on the functionality of specific T cells, (ii) to evaluate the impact of prevaccine immunization, by comparing the postvaccinal response in older adults without and in older adults with prior COVID-19, and finally (iii) to evaluate the impact of frailty, nutrition, and immunosenescence features on postvaccination immune response.

## Material and Methods

### Study Design and Participants

This was a prospective single-center study conducted at the Lille University Hospital, in the North of France. Participants were consecutively included in the study and were healthcare workers (HCW; hereafter referred to as young adults) aged 18–65 years and LCTF residents (hereafter referred to as older residents or older adults) aged >65 years who consented to be vaccinated with BNT162b2 mRNA vaccine and were willing to comply with the study procedures. None of the enrolled participants had a recent, current, or persistent infectious disease, any neoplasia diagnosis in the last 5 years, or treatment with steroids and/or immunosuppressants. Participant characteristics collected at baseline included confirmation of prior SARS-CoV-2 infection, determined by polymerase chain reaction (PCR) and/or high antibody titer to SARS-CoV-2 spike S1 domain: participants with a history of positive PCR and/or who tested positive for anti-S1 antibodies were considered COVID-19-recovered, and the other participants as “COVID-19-naive.” Among older adults, Geriatric Nutritional Risk Index was calculated according to the Lorentz formula: GNRI = (1.489 × albumin, g/l) + (41.7 × present/ideal body weight), with the ideal weight calculated according to the Lorentz formula (8). Frailty was assessed with the Clinical Frailty Scale as proposed by Rockwood et al. (9) and using the Fried frailty phenotype criteria (10). All participants received the two-dose BNT162b2 vaccination at a 3-week dosing interval: the first dose was administered at Day 0 (D0) and the second dose between D21 and D28. Serum samples were collected for all participants at D0, and D90 (± 14 days) after the first dose.

### Anti-SARS-CoV-2 Antibodies

Anti-SARS-CoV2 spike S1 domain-specific immunoglobulin G (IgG) was assessed in serum samples using ELISA (Quantivac, Euroimmun Lübeck, Germany), with a sensitivity of 90.3 and a specificity of 99.8% according to the manufacturer’s data. The maximum IgG level that could be determined with appropriate precision after dilution was 1,920 relative units per milliliter (RU/ml).

### SARS-CoV-2 Neutralization Assay

Neutralizing antibodies were investigated using a live virus neutralization assay (LV-NT). A classical B.1.1.7 lineage (20I/501Y.V1) SARS-CoV-2 strain, previously isolated from a clinical specimen and propagated in Vero E6 cells, was used in all experiments. The whole genome sequence of the viral isolate was submitted to GISAID (accession reference EPI_ISL_1653931). In brief, serial twofold dilutions (starting from 1:10) of the heated serum (56°C for 30 min) were incubated for 1 h at 37°C with a viral solution containing 100 TCID50 of SARS-CoV-2 and then added to Vero E6 cell monolayers in a 96-well plate. The cytopathic effect was recorded after 3 days, and the serum virus neutralization titer (V-NT50) was defined as the reciprocal value of the highest dilution that showed at least 50% protection of cells. A sample with a titer ≥20 was defined as positive. Negative signals were set to 0 for statistical analyses.

### SARS-CoV-2 Pseudovirus Neutralization Assay

To further assess the neutralizing activity of sera, retroviral pseudoparticles containing the SARS-CoV-2 glycoprotein S (SARS-CoV-2pp) were produced as previously described (11), with a plasmid encoding the human “codon-optimized” sequence of the SARS-CoV-2 glycoprotein spike (accession number: MN908947). The supernatants containing the SARS-CoV-2pp were harvested at 48-h posttransfection and filtered through a 0.45-µm membrane and stored at −80°C. The serum neutralization test was performed as previously described (12). In brief, 20 µl of SARS-CoV-2pp were incubated in the diluted serum at a final volume of 50 µl of DMEM+Glutamax+penicillin-streptomycin+10% fetal calf serum (FCS) for 30 min at room temperature. The mixture was then added to HEK 293TT-ACE2 plated the day before (HEK 2932TT cells stably expressing the hACE2 receptor are seeded at 4,500 cells/well in a volume of 50 µl of DMEM+Glutamax+penicillin-streptomycin+10% FCS mixture) (13). At 48-h postinfection, Luciferase activity was measured using the Luciferase Assay System kit (Charbonnières-les-Bains, Promega FR, Charbonnières-les-Bains, France) as recommended by the manufacturer and expressed as relative luciferase units (RLUs). RLUs were compared and normalized with the wells where pseudoparticles were added in the absence of serum (100%). Serum pseudovirus neutralization titer 50 (PV-NT50) was expressed as the maximal dilution of the sera where the reduction of the signal is greater than 50%. The titer was multiplied by 781, since the initial volume of the sera tested was 8 µl and had to be normalized to 1 ml (14).

### Peripheral Blood Mononuclear Cells Preparation

Isolation and numeration of peripheral blood mononuclear cells (PBMCs) were performed from 10 to 15 ml of freshly collected heparinized blood samples. In brief, T-cell Xtend (Oxford Immunotec, Abingdon, UK) at a concentration of 25 µl/ml of blood was added 15 min prior to isolation to remove cell debris and aggregates. SepMate-50 ml (StemCell Technologies, Vancouver, Canada) was then used for density gradient centrifugation. PBMCs were collected and washed twice using RPMI. Isolated cells were suspended in AIM-V medium and counted using flow cytometry with CD45 staining (Beckman Coulter, Brea, CA, USA) and Flow-Count Fluorospheres (Beckman Coulter). Normalization of the cell suspension was performed at a final concentration of 2.5.10^6^ cells/ml for T-CoV-Spot assay and 10.10^6^ cells/ml for flow cytometry analyses.

### IFNγ ELISpot Assay—T-CoV-Spot Assay

T-CoV-Spot assay was performed as previously described (8). In brief, overlapping peptide pools covering the N-terminal S1 domain were used (PepTivator_SARS-CoV-2, Miltenyi Biotec, Bergisch Gladbach, Germany). Peptides consisted of 15-mer sequences with 11 amino acids overlap. Microtiter plates coated with anti-IFNγ antibodies (T-SPOT.TB, Oxford Immunotec) were used. The cell suspension was normalized at a final concentration of 2.5 × 10^6^ cells/ml, and plating with SARS-CoV-2 antigens was manually performed (2.5 × 10^5^ PBMCs added per well). Peptide pools were added at a concentration of 0.5 μg/ml. Following an incubation at 37°C for 16–20 h in a humidified atmosphere containing 5% CO_2_, wells were washed and incubated with conjugate reagent for 1 h at 2°C–8°C. After a washing step, wells were developed for 7 min with substrate solution. The reaction was stopped by adding distilled water. Plates were allowed to dry in an oven at 37°C for 1 h. Spot-forming cells (SFCs) were detected using the CTL ImmunoSpot plate reader. Appropriate negative and positive controls were used (15).

### Flow Cytometry Analyses

In addition to IFNγ-secreting cells by ELISpot, SARS-CoV-2-specific T-cell detection was also analyzed using flow cytometry. PBMC suspensions were normalized at a final concentration of 10 × 10^6^ cells/ml and 1 × 10^6^ cells were incubated in RPMI for 16–20 h at 37°C in a humidified atmosphere containing 5% CO_2_. Then, 7-amino-actinomycin D (7AAD) (BioLegend, San Diego, CA, USA), Pacific Blue-conjugated anti-CD107a antibody (clone H4A3; Beckman-Coulter) and the same peptide pools, at the same concentrations than for the ELISpot assay, were added to the cell suspension for 1 h (37°C, 5% CO_2_). Brefeldin A (Sigma-Aldrich, St. Louis, MO, USA) and monensin (BioLegend) were added at 2.5 µm/ml and 2 µM, respectively. The obtained cell preparation was conserved for 4 h (37°C, 5% CO_2_). The washed cells were then permeabilized with Cytofix/Cytoperm™ Fixation/Permeabilization Kit, according to the manufacturer recommendations (Becton Dickinson), and two washing steps with perm/wash buffer were performed (Beckton Dickinson). For detection of surface molecules, antibodies against CD3 (APC-Alexa750 conjugated, clone UCHT1, Beckman Coulter), CD4 (APC, clone 13B8.2, Beckman Coulter), CD8 (Alexa700, clone B9.11, Beckman Coulter), CD154 (PE, clone TRAP-1, Beckman Coulter, IM2216U), and CD69 (FITC, clone FN50, BioLegend, catalog no 310904) were used. Intracellular cytokines were detected with antibodies against TNFα (PC7, clone Mab11, BioLegend), IL-2 (BV605, clone MQ1-17H12, Biolegend), and IFNγ (BV650, clone 4S.B3, BioLegend). Each cell preparation was totally analyzed (around 300,000 T cells). For assessment of whole blood-naive/memory T cells at baseline in LTCF residents, antibodies against CD4 (Pacific Blue, clone 13B8.2, Beckman Coulter), CD8 (APC, clone B9.11, Beckman Coulter, catalog No. A99023), CD45RA (FITC, clone 2H4, Beckman Coulter), and CCR7 (PE, clone G043H7, Beckman Coulter) were used. Cells were analyzed on a Cytoflex S (Beckman Coulter) flow cytometer.

### Fluorescence-Activated Cell Sorter Data Analysis

Fluorescence-activated cell sorter (FACS) data were analyzed with Kaluza Analysis Software (Beckman Coulter). The gating strategy for analysis of antigen-specific T cells is illustrated in the **Supplementary Figure S1**. For the activation-induced marker (AIM) T-cell assay, a specific T-cell response was considered positive when the stimulation index was 2 or higher, i.e., when the antigen-stimulated cultures contained at least twofold higher frequencies of CD154^+^CD69^+^ cells among alive (7-AAD−) CD4^+^ T cells (AIM^+^CD4^+^ T cells), or CD107a^+^CD69^+^ cells among alive CD8^+^ T cells (AIM^+^CD8^+^ T cells), compared with the unstimulated control sample. No further background subtraction was applied. Coexpression of intracellular cytokines was assessed among AIM^+^CD4^+^ and CD8^+^ T cells using a Boolean gating strategy. Unsupervised analysis was conducted using t-distributed stochastic neighbor embedding (t-SNE) in AIM^+^CD4^+^ or CD8^+^ T cells (Cytobank, Beckman Coulter). All datasets were extracted from the pregating made with Kaluza on AIM^+^ T cells, group concatenations were made, and all data were imported into Cytobank. Unsupervised cell subset identification (clustering) was also performed for analysis of cytokine productions by AIM^+^CD4^+^ and CD8^+^ T cells. Percentages of each main subsets of specific T cells (according to production of 0/1, 2, or 3 cytokines) obtained by the unsupervised FlowSOM analysis (considered the addition of all cluster abundance in the subset) were reported on the subsets (Cytobank, Beckman Coulter).

### Cytokine Measurements

Plasma IL‐1β, IL‐6, TNFα, and IL‐10 concentrations were assessed using the Ella Automated Immunoassay System (ProteinSimple, San Jose, CA, USA) following the manufacturer’s recommendations.

### Statistical Analyses

Categorical variables are expressed as numbers (percentages) and quantitative variables are expressed as median (interquartile range). Normality distribution was assessed graphically and using the Shapiro-Wilk test. Immune parameters were compared within the same group between the baseline and 3-month assessments using the Wilcoxon signed rank test. Comparisons of immune parameters between the four study groups (COVID-19-naive older, COVID-19-recovered older, COVID-19-naive young, and COVID-19-recovered young) were done using the Kruskal-Wallis test followed by *post-hoc* Dunn’s tests for quantitative measures and chi‐squared test (or Fisher’s exact test in cases of expected cell frequency <5) for responder rates. Comparisons of baseline characteristics in COVID-19-recovered older adults and D90 characteristics in COVID-19-naive older adults (natural post-COVID-19 *versus* post-BNT162b2 immunization) were done using the Mann-Whitney *U* test. We assessed the correlation between age, vaccinal response parameters, nutritional, frailty, or immunosenescence parameters by calculating Spearman’s rank correlation (*r*) coefficients, with their 95% confidence intervals based on the Fisher Z-transformation. Statistical tests were done at the two-tailed α level of 0.05. No correction for multiple testing was carried out. Data analyses and graphs were performed using the GraphPad Prism software version 9.1.2 (GraphPad Software, La Jolla, CA, USA).

### Ethics

This study was performed in accordance with the Declaration of Helsinki principles for ethical research. The study was approved by the Ile-De-France V (ID‐CRB 2021-A00119-32) ethics committee. All participants (and/or their legal representative if required) received detailed information and signed a consent form before participating in the study. The study was registered in ClinicalTrials.gov, with the identifier NCT04760704.

## Results

### Immunogenicity in COVID-19-Naive Young and Older Subjects

**Figure 1:**
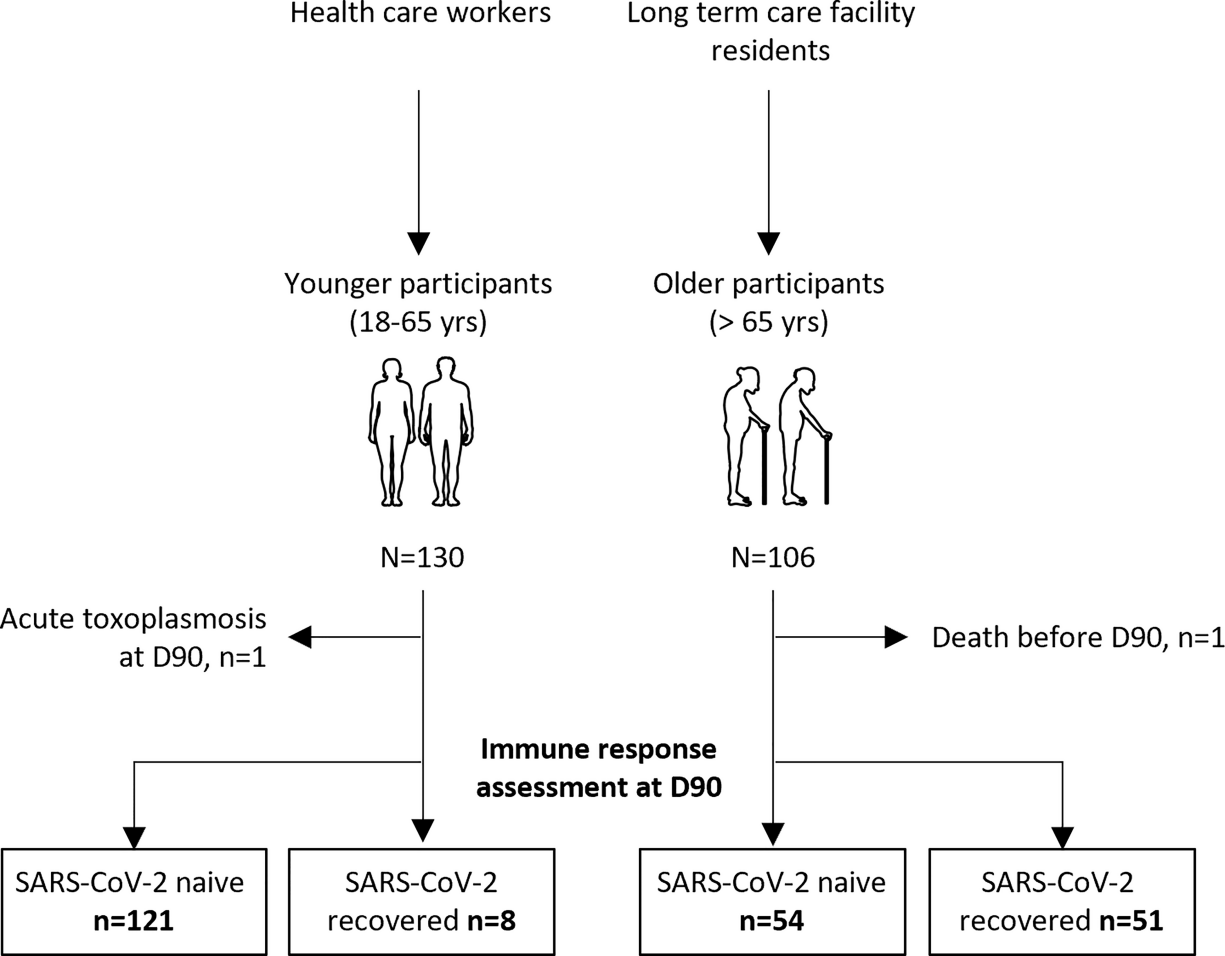
Flow chart of the study.

**Figure 2:**
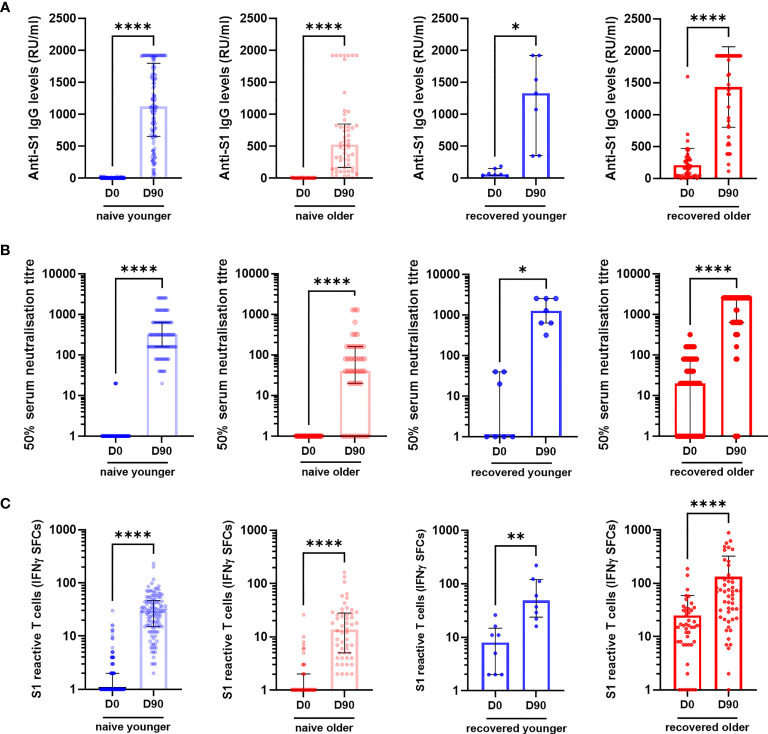
Specific antibody and T-cell responses in older and in young adults before (D0) and 3 months (D90) after the first injection of BNT162b2. **(A)** Anti-S1 IgG, **(B)** serum neutralization assay against live virus, and **(C)** S1-reactive T cells (ELISpot) in COVID-19-naïve and in COVID-19-recovered participants. Wilcoxon matched-pairs signed rank test was used for paired comparisons. ^*^ *p*-values < 0.05; ^**^ *p*-values < 0.01; ^****^ *p*-values < 0.0001. CTL, IFNγ SFCs, interferon gamma spot-forming cells.

We consecutively included 130 young adults (median [interquartile range (IQR)] age, 44.0 years [39.7; 50.5]) and 106 older residents (median [IQR] age, 86.5 years [81.0; 90.0]) who had received two vaccine doses (**Table 1**). Participants were sampled before (D0) and 90 days (D90) after the first dose: 129 young adults and 105 older residents had both samples (**Figure 1**). In young and older subjects, with or without prior COVID-19, anti-S1 IgG, neutralizing antibodies, and IFNγ-secreting T-cell levels increased from D0 to D90 (**Figure 2** and **Supplementary Figure S1**).

**Table 1:** Characteristics of healthcare workers (young adults) and long-term care facility residents (older adults) enrolled.

Characteristics	Young (*n* = 130)	Older (*n* = 106)
**Age (years), median [IQR]**	44 [39.5; 50.5]	86.5 [81.0; 90.0]
**Female [*n* (%)]**	96 (73.9)	74 (69.8)
**Comorbidities [*n* (%)]**		
** Hypertension**	1 (0.8)	70 (66.0)
** Coronary heart disease**	0	73 (68.9)
** Diabetes**	1 (0.8)	22 (20.7)
** COPD**	0	25 (23.6)
** Chronic renal failure**	0	29 (27.3)
** Dementia**	na	95 (89.6)
**Prior COVID-19**	8 (6.1)	51 (48.1)
** Asymptomatic [*n* (%)]**	8 (6.1)	8 (15.7)
** Mild disease [no oxygen requirement; *n* (%)]**	0	30 (58.8)
** Moderate disease [oxygen requirement; *n* (%)]**	0	10 (17.2)
** Severe/critical disease [high-flow ventilation, OTI; *n* (%)]**	0	3 (5.9)
** Time from infection diagnosis to first BNT162b2 injection [months; median (IQR)]**	0	4.2 [3.3–8.3]
**Nutritional status [median (IQR)]**		
** Albuminemia** **^a^** **(g/l)**	na	34 [31.0; 37.5]
** Vitamin D (IU/l)** **^a^**	na	30 [27.0; 36.0]
** Body weight (kg)**	na	60 [51.0; 72.0]
** Body mass index (kg/m^2^)**	na	23 [20.0; 27.0]
** Geriatric Nutritional Risk Index** **^a^**	na	96.1 [86.4; 104.4]
**Frailty [median (IQR)]**		
** Clinical Frailty Scale**	na	7 [7; 8]
** Fried frailty phenotype criteria**	na	4 [3; 4]

COPD, chronic obstructive pulmonary disease; na, not available; OTI, orotracheal intubation.

Continuous data are given as median [IQR]; categorical data are given as numbers (%).

a Data were missing for 17 older adults.

**Figure 3:**
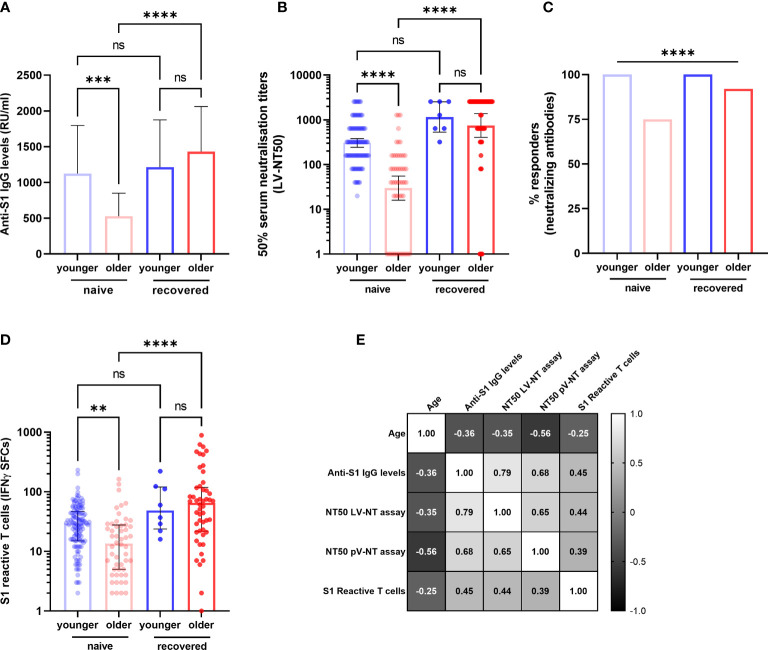
Specific antibody and T-cell responses in older and in young adults 3 months after the first injection of BNT162b2. **(A)** Antibody responses assessed by ELISA (anti-S1 IgG) (COVID-19-naive younger *n* = 121, COVID-19-naive older *n* = 54, COVID-19-recovered young *n* = 8, COVID-19-recovered older *n* = 47; median [interquartile range (IQR)] are shown). **(B)** Serum neutralization assay against live virus (COVID-19-naive young adults *n* = 101, COVID-19-naive older *n* = 52, COVID-19-recovered young *n* = 7, COVID-19-recovered older *n* = 51; geometric median and 95% confidence interval are shown). **(C)** Participants with detectable neutralizing antibodies according to live virus-neutralizing assay (titer ≥1:20). **(D)** Number of S1 peptide pool reactive T cells (ELISpot) (COVID-19-naive young adults *n* = 121, COVID-19-naive older *n* = 52, COVID-19-recovered young *n* = 8, COVID-19-recovered older *n* = 50; median [interquartile range (IQR)] are shown). **(E)** Correlations between age and main immune parameters of the postvaccinal response at 3 months in COVID-19-naive older and COVID-19-naive young adults. Values are Spearman’s rank correlation (r) coefficients. The number of pairs that were analyzed, *p*-values and 95% confidence intervals of significant correlations are detailed in **Table 2**. ^**^ *p*-values <0.01; ^***^ *p*-values < 0.001; ^****^ *p*-values < 0.0001; ns, not significant. IFNγ SFCs, interferon gamma spot-forming cells; LV-NT50, 50% serum neutralization titer in live virus neutralization assay; pV-NT50, 50% serum neutralization titer in pseudovirus neutralization assay.

Our primary objective was to compare the specific memory response in COVID-19-naive younger (*n* *=* 121/129) and in COVID-19-naive older adults (*n* *=* 54/105). At D90, S1 IgG reactivity was detected in almost all participants in both groups (99.2% of younger and 97.2% of older adults), but the median titer of anti-S1 IgG antibodies was two times lower among the older residents (*p* < 0.001) (**Figure 3A**). The difference was greater for neutralizing antibodies, with a geometric mean of 50% serum neutralization titer (NT50) 10.2 times lower in the older group according to the LV-NT assay (**Figure 3B**) (mean [95% confidence interval (CI)] 29.8 [16.0; 55.2] versus 305.0 [243.1; 382.6]; median [IQR] titers: 40.0 [5.7;160.0] versus 320.0 [160.0; 640.0], *p* < 0.0001). The number of responders (i.e., participants who had detectable neutralizing antibodies) was 39 (*n* = 51 available data, 76.5%) COVID-19-naive older adults and 101 (*n* *=* 101 available data, 100%) (*p* < 0.0001) COVID-19-naive younger adults (**Figure 3C**). The mean NT50 in each group was consistent with the PV-NT50 assay (**Supplementary Figure S2**). Regarding cellular response, T cells reactive to the S1 subunit detected by ELISpot were less frequent in the older than in the younger group (13.5 [25.0–27.57] versus 29.5 [15.0; 46.5], respectively) (*p* = 0.002) (**Figure 3D**). To confirm our results, all acquired parameters were correlated with age in COVID-19-naive young and older participants. Age negatively correlated with anti-S1 IgG, neutralizing antibody titers, and count of specific IFNγ-secreting T cells, which support the differences observed between the two groups (**Figure 3E**). There were also strong positive correlations between the immune parameters, which highlights both the conserved links between these different adaptive responses among the older population, and the robustness of the chosen approaches (**Figure 3E** and **Table 2**).

**Table 2:** Correlations between age and main immune parameters of the postvaccinal response at 3 months in COVID-19-naive young and older participants.

Correlation	Spearman’s rank (*r*)	*p*-value	95% CI	Sample size
**Age with**				
** Anti-S1 IgG levels**	−0.36	<0.0001	−0.48; −0.22	181
** NT50 LV-NT**	−0.35	<0.0001	−0.48; −0.19	146
** NT50 PV-NT**	−0.56	<0.0001	−0.66; −0.44	160
** S1-reactive T cells (ELISpot)**	−0.25	0.0008	−0.38; −0.10	179
**Anti-S1 IgG antibodies with**				
** NT50 LV-NT**	0.79	<0.0001	0.72; 0.85	181
** NT50 PV-NT**	0.68	<0.0001	0.58; 0.75	146
** S1-reactive T cells (ELISpot)**	0.45	<0.0001	0.32; 0.56	160
**NT50 LV-NT with**				
** NT50 PV-NT**	0.65	<0.0001	0.53; 0.74	127
** S1-reactive T cells (ELISpot)**	0.44	<0.0001	0.29; 0.56	145
**NT50 PV-NT with**				
** S1-reactive T cells (ELISpot)**	0.39	<0.0001	0.25; 0.52	158

All correlations are presented in **Figure 3E** (with Spearman’s rank correlation (r) coefficients). NT50 LV-NT assay, 50% serum neutralization titer in live virus neutralization assay; NT50 PV-NT assay, 50% serum neutralization titer in pseudovirus neutralization assay; CI, confidence interval.

**Figure 4:**
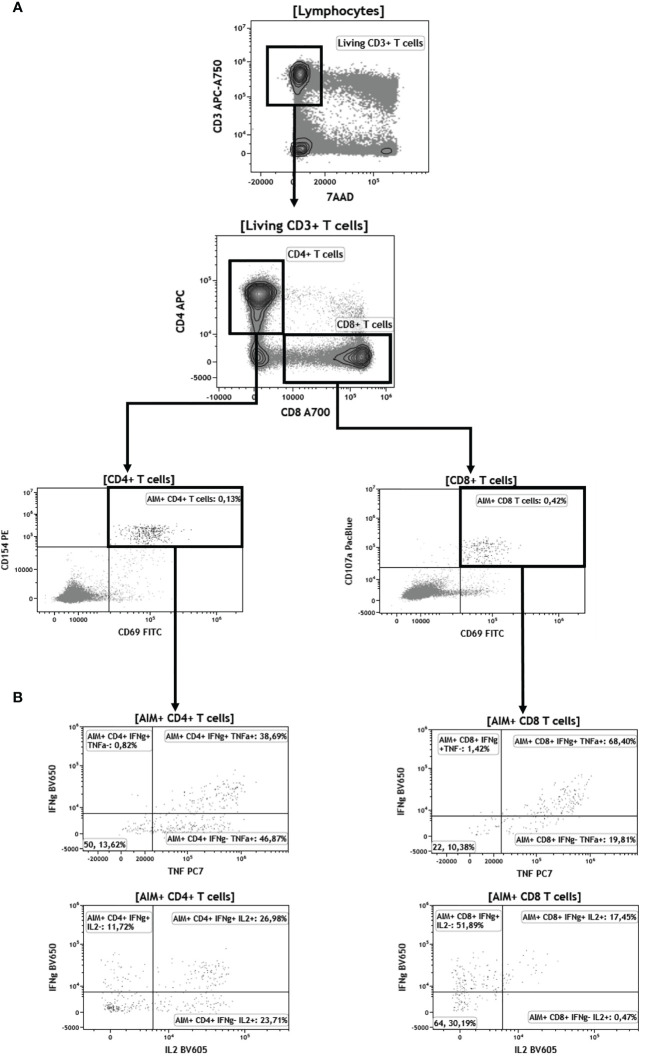
Gating strategy for flow cytometry analyses of CD4^+^ and CD8^+^ T cells after BNT162b vaccination. **(A)** Identification of activation induced markers (AIM^+^ cells). Briefly, “living CD3^+^ T cells” are identified as 7-aminoactinomycine D (7AAD)-negative and CD3-positive cells. Among this population, CD4^+^ and CD8^+^ T cells are selected according to CD4^+^ and CD8^+^ expression, respectively. AIM^+^ cells among CD4^+^ T cells are both CD154^+^ and CD69^+^. AIM^+^ cells among CD8^+^ T cells are both CD107a^+^ and CD69^+^. **(B)** Representative plots displaying IFNγ, IL-2, and TNFα expression among AIM^+^CD4^+^ and CD8^+^ T cells.

**Figure 5:**
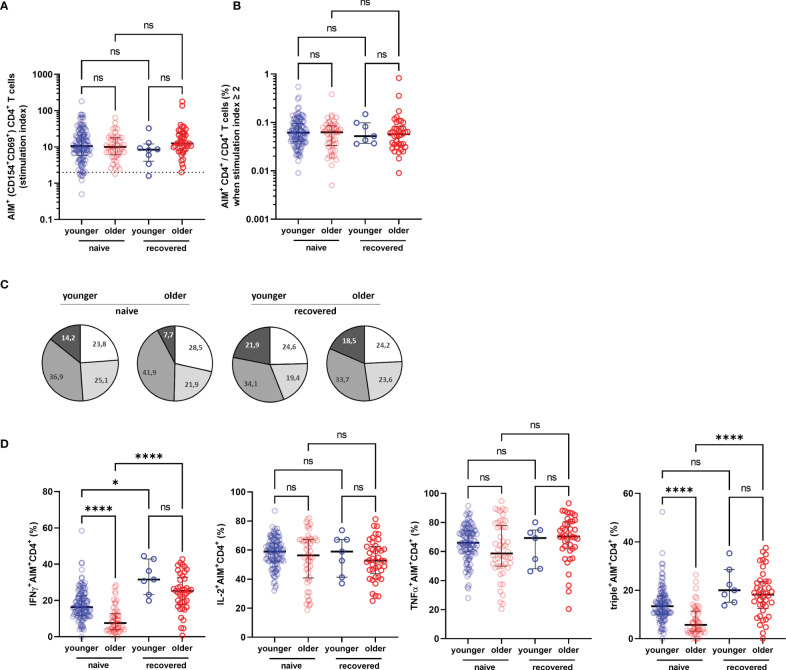
Specific CD4^+^ T-cell response in older and in young adults 3 months after the first injection of BNT162b2. **(A)** Specific CD4^+^ T cells according to activation-induced markers (AIM), reported with their stimulation index. **(B)** Percentage of AIM^+^CD4^+^ T cells among total CD4^+^ T cells in responders, i.e., participants with a stimulation index ≥2. **(C)** Pie charts representing the relative proportions of AIM^+^CD4^+^ T cells producing none (white), one (light grey), two (medium grey), or three cytokines (dark grey) out of INFγ, IL-2, and TNFα according to participant group and past history of COVID-19 (naive and recovered). **(D)** Proportion of AIM^+^CD4^+^ cells producing IFNγ, IL-2, TNFα, and proportion of IFNγ^+^IL-2^+^TNFα^+^ (triple^+^) CD4^+^ T cells according to participant group and past history of COVID-19 (naive and recovered). (COVID-19-naive young adults *n* = 113, COVID-19-naive older *n* = 48, COVID-19-recovered young *n* = 8, COVID-19-recovered older *n* = 41; median [interquartile range (IQR)] are shown). ^*^ *p*-values < 0.05; ^****^ *p*-values < 0.0001; ns, not significant. AIM^+^, cell-expressing activation-induced markers.

**Figure 6:**
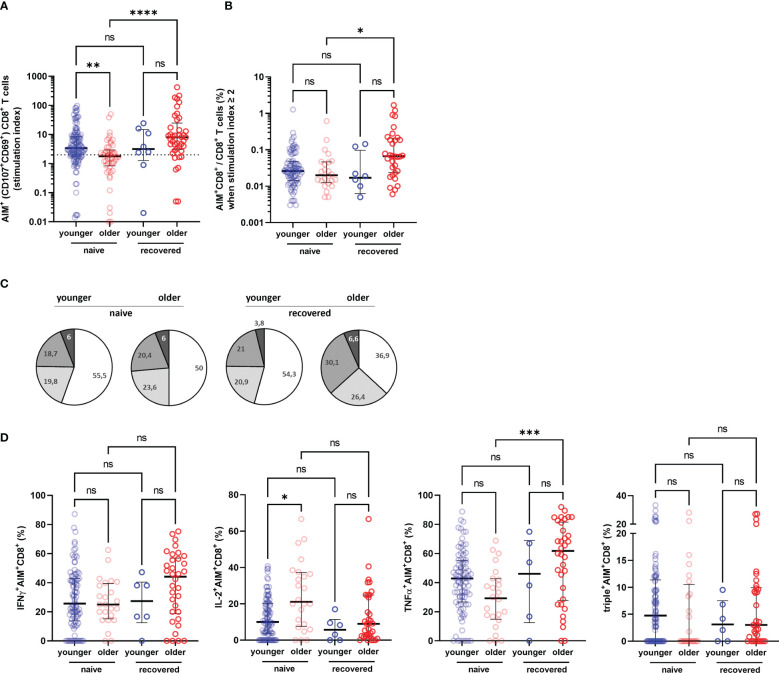
Specific CD8^+^ T-cell response in older and in young adults 3 months after the first injection of BNT162b2. **(A)** Specific CD8^+^ T cells according to activation-induced markers (AIM), reported with their stimulation index. **(B)** Percentage of AIM^+^CD8^+^ T cells among total CD8^+^ T cells in responders, i.e., participants with a stimulation index ≥2. **(C)** Pie charts representing the relative proportions of AIM^+^CD8^+^ T cells producing none (white), one (light grey), two (medium grey), or three cytokines (dark grey) out of INFγ, IL-2, and TNFα according to participant group and past history of COVID-19 (naive and recovered). **(D)** Proportion of AIM^+^CD4^+^ cells producing IFNγ, IL-2, TNFα, and proportion of IFNγ^+^IL-2^+^TNFα^+^ (triple^+^) CD4^+^ T cells according to participant group and past history of COVID-19 (naive and recovered). (COVID-19-naive young adults *n* = 113, COVID-19-naive older *n* = 48, COVID-19-recovered young *n* = 8, COVID-19-recovered older *n* = 41; median [interquartile range (IQR)] are shown). ^*^ *p*-values < 0.05; ^**^ *p*-values < 0.01; ^***^ *p*-values < 0.001; ^****^ *p*-values < 0.0001; ns, not significant. AIM^+^, cell-expressing activation-induced markers.

To better assess the functionality of specific T cells acquired after vaccination, we have distinguished S1-specific CD4^+^ and CD8^+^ T cells by quantification of cells expressing the surface activation-induced markers (AIM^+^ T cells) after stimulation with S1 overlapping peptide pools (subjects with a stimulation index >2 were regarded responders), and the percentage of CD4^+^ or CD8+ T cells able to produce one, two, and/or three cytokines among INFγ, IL-2, and TNFα (polyfunctional cells) (**Figure 4**). We observed no significant difference in the rate of participants with detectable AIM^+^CD4^+^ T cells between the COVID-19-naive young and older adults according to stimulation index (89.8% versus 97.9%, respectively, *p* > 0.05) and similar frequency of AIM^+^CD4^+^ among CD4^+^ T cell in both groups (**Figures 5A, B**). Conversely, the frequencies of CD4^+^ effector cells differed (**Figure 5C**): the amount of specific AIM^+^IFNγ^+^ and AIM^+^IFNγ^+^IL-2^+^TNFα^+^ (triple^+^) CD4^+^ T cells were lower in COVID-19-naive older participants than in COVID-19-naive young adults (**Figure 5D**). Of note, AIM^+^IFNγ^+^CD4^+^ T-cell counts detected by flow cytometry correlated very well with IFNγ-secreting T-cell counts in ELISpot, confirming the robustness of our ELISPot assay to assess the T-cell response (Spearman’s coefficient *r* [95% CI] = 0.47 [0.34–0.59]; *p* < 0.0001; available pairs: *n* = 151). Regarding anti-S1-specific CD8^+^ T cells, more COVID-19-naive young participants developed AIM^+^CD8^+^ T cells than COVID-19-naive older adults (76.4% versus 48.0%, respectively, *p* *=* 0.0018) (**Figure 6A**), but the frequency of AIM^+^CD8^+^ among total CD8^+^ T cells was similar in responders of both groups (**Figure 6B**). We also noted that, in the COVID-19-naive population, the frequency of AIM^+^IL-2^+^CD8^+^ T cells was greater among the older than among the younger, but frequencies of IFNγ^+^, TNFα^+^, or triple^+^ CD8^+^ T cells were not different (**Figures 6C, D**).

### Immunogenicity of BNT162b Vaccine in COVID-19-Naive and COVID-19-Recovered Older People

Our secondary objective was to evaluate the capacity of the vaccination to boost the natural anti-SARS-CoV-2 memory response. Among the included participants, 51 COVID-19-recovered older adults (*n* = 5 according to high anti-S1 IgG titers, *n* = 46 by positive PCR: median [IQR] interval 4.2 months [3.3–8.3]) were compared with COVID-19-naive counterparts (*n* = 54): 92.2% of COVID-19-recovered older adults produced detectable neutralizing antibodies compared with 76.5% of COVID-19-naive older participants (**Figure 3C**). In addition, after two vaccine doses, the anti-S1 IgG (**Figure 3A**), the neutralizing antibody levels (**Figure 3B**), IFNγ-secreting T-cell counts in ELISpot (**Figure 3D**), AIM^+^IFNγ^+^CD4^+^ and AIM^+^triple^+^CD4^+^ frequencies (**Figures 5C, D**), total AIM^+^CD8^+^T cells (**Figure 6B**), and AIM^+^TNFα^+^CD8^+^ T-cell frequencies (**Figures 4C, D**) increased to a greater extent among COVID-19-recovered older adults compared with COVID-19-naive older adults.

**Figure 7:**
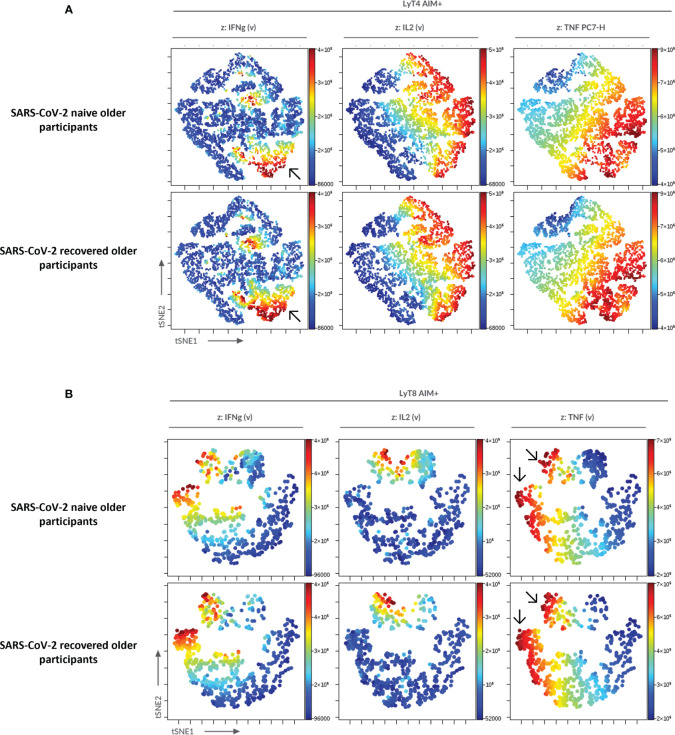
Unsupervised analysis of CD4^+^ and CD8^+^ T-cell functionality in older participants using t-distributed stochastic neighbor embedding (t-SNE). AIM^+^CD4^+^ **(A)** and AIM^+^CD8^+^ **(B)** T cells from older participants were concatenated and subjected to unsupervised analysis using t-SNE; highlighted (z-dimension) are areas with IFNγ, IL-2, or TNFα cell expression in COVID-19-naive and COVID-19-recovered older adults. To be noted, the higher frequency of IFNγ^+^CD4^+^ T cells and of TNFα^+^CD8^+^ T cells in COVID-19-recovered older adults (arrows).

To confirm the significance of our results in specific cellular responses, we used an automatized cluster analysis of T-cell subsets (**Supplementary Figure S3**). The hierarchical clustering confirmed the lower cytokine production in COVID-19-naive older participants compared with the three other groups of interest. The cluster analysis also showed a higher CD8^+^/CD4^+^ ratio among AIM^+^ T cells in the COVID-19-recovered older group. Similarly, an unsupervised analysis using t-SNE corroborated the reduced cytokine production in COVID-19-naive compared with the COVID-19-recovered older participants (**Figure 7**).

### Immunogenicity of BNT162b Vaccine According to Frailty, Nutritional, and Immunosenescence Parameters in COVID-19-Naive Older People

**Figure 8:**
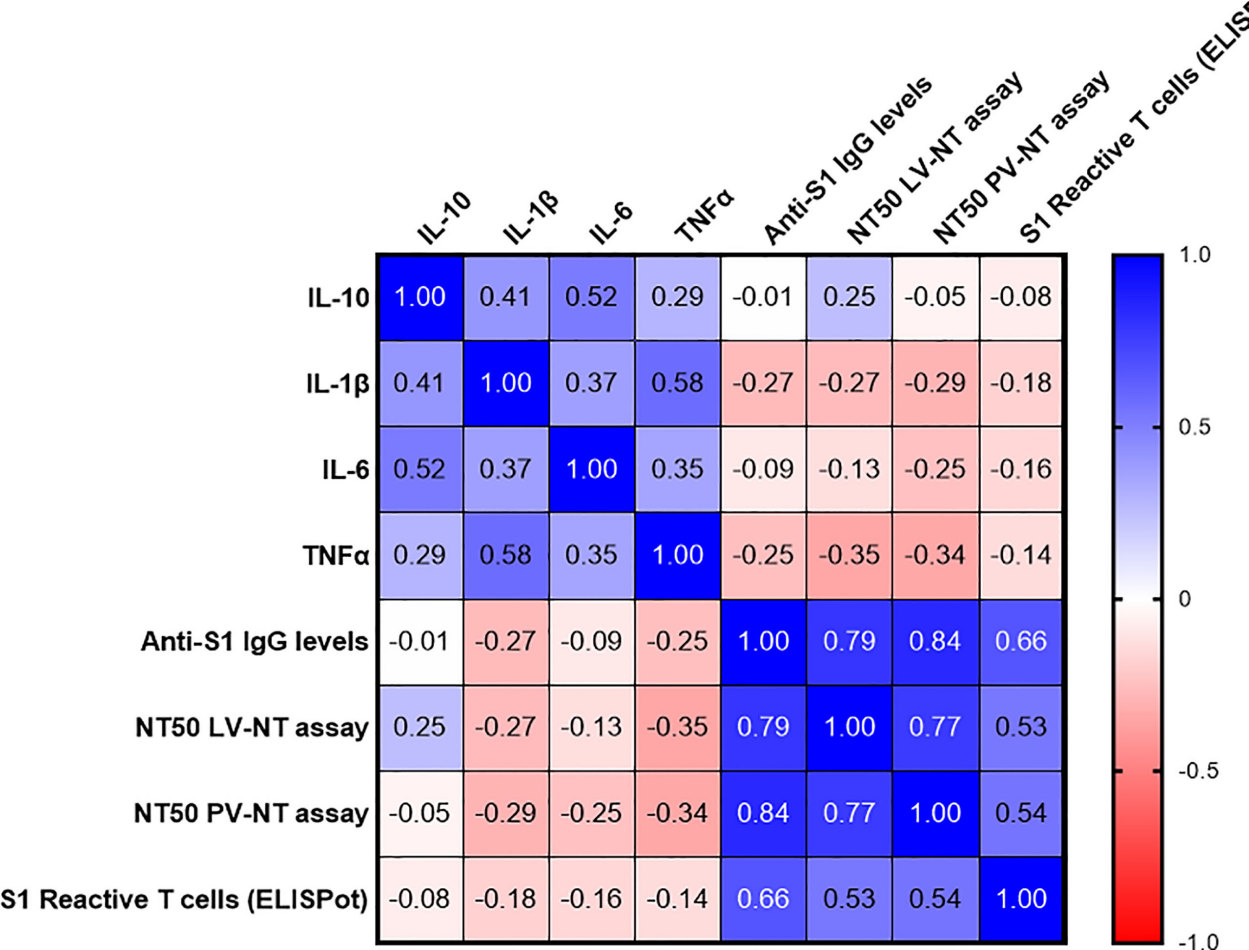
Correlations between plasma cytokines levels at baseline, and main immune parameters of the postvaccinal response at 3 months in COVID-19-naive older subjects. The values correspond to Spearman’s rank correlation (*r*) coefficients. Only two correlations were found to be significant, between TNFα levels and NT50 LV-NT (*r* (95% CI) −0.35 [−0.62; 0.007], *p* = 0.048, sample size *n* = 33) and between TNFα levels and NT50 pV-NT (*r* (95% CI) −0.34 [−0.60; 0.02], *p* = 0.034, sample size *n* = 38). AIM^+^, cell-expressing activation-induced markers; NT50 LV-NT assay, 50% serum neutralization titer in live virus neutralization assay; NT50 PV-NT assay, 50% serum neutralization titer in pseudovirus neutralization assay.

Finally, we evaluated whether some frailty, nutritional, and immunosenescence parameters could account for the poor vaccinal response among the COVID-19-naive older adults. As other authors (9), we failed to identify any clear link between vaccinal response and frailty, nutritional state (**Supplementary Figure S4**), or baseline B cell, total T-cell, and naive T-cell counts (**Supplementary Figure S5**). We also assessed plasma levels of three major proinflammatory cytokines, IL-1β, IL-6 and TNFα, and IL-10, an anti-inflammatory cytokine, in COVID-19-naive older adults. Plasma IL-1 β levels tended to be negatively correlated with anti-S1 IgG and live virus-neutralizing antibodies. Plasma TNFα levels correlated negatively with both neutralizing titers (**Figure 8**).

## Discussion

Our work demonstrates that COVID-19-naive older adults have a poor memory immune response to BNT162b2 mRNA vaccine compared with the younger adults. Considering the impact of COVID-19 on life expectancy in LCTF residents (16), specific vaccinal strategy may be required in this frail population.

Our results are in line with earlier evaluations of antibody response after a first dose (9, 10) and between 14 and 28 days after the second one (17–20), indicating a poorer response in the older people. Even though we did not assess memory-switched B cells, our results obtained 90 days after the first dose and 60 days after the second dose may reflect the memory response established after vaccination rather than a response being initiated and may predict that immunity may wane even more over time.

Our study also brings to light new elements on the function of T cells in the older population after two doses of BNT162b2. As in previous works using FluoroSpot or ELISpot T-cell assays (18, 20, 21), we also reported here an impaired specific T-cell response after a full vaccination scheme in older people. Using flow cytometry, we observed that specific IFNγ^+^ and triple^+^CD4^+^ T cells, the most important subsets in the orchestration of the whole adaptive immune response, were lower in COVID-19-naive older participants than in COVID-19-naive young adults. We also noted that, in the COVID-19-naive population, the frequency of AIM^+^IL-2^+^CD8^+^ T cells (but not IFNγ^+^ or TNFα^+^CD8^+^ T cells) was greater among the older subjects compared with the young group. This suggests that, while CD8^+^ T cells can be highly activated with SARS-CoV-2 antigens in the naive older population, the main effector cytokines required for antiviral response are not produced.

Our study also demonstrated that specific antibody response is greater in COVID-19-recovered older residents (compared with COVID-19-naive), and at a level similar to that of young participants. These results are in agreement with previous reports suggesting that patients with prior COVID-19 infection had a better antibody response, regardless of the age (21–23). Our work also shows that specific IFNγ^+^ and triple^+^CD4^+^ T cells, and specific TNFα^+^CD8^+^ T cells, the latter being the most important in antiviral defense, were also highly boosted in participants who had a prior COVID-19 infection. Considering the major role of these cell subsets in limiting the disease severity (23, 24), a repeated vaccination could be effective in increasing the immune response in the older population.

We also observed that “inflammageing” may play a role in the poor anti-SARS-CoV-2 antibody response in the older, and particularly TNFα. This is in line with previous data in human and mice models, which reported that serum TNFα negatively correlated with the B-cell response and a vaccine-specific antibody response (24).

### Strengths and Limitations

This study is the first one to assess the functionality of specific T cells in older people after two doses of BNT162b: our results obtained by flow cytometry support the results obtained by ELISpot and brings new elements about the quality of the postvaccination T-cell immune response in this at-risk population for severe COVID-19. We are also able to discuss the higher immunogenicity of BNT162b vaccine in older people with previous COVID-19, not only considering the antibody response, but also the T-cell response and its functionality.

Our study is, however, limited in that, due to the recommendations applied in France at the time of the study, we were unable to assess whether a single dose of this vaccine after exposure to COVID-19 would have generated a sufficient response in these individuals. Also, the relatively short follow-up period only allowed us to assess the short-term effects of the vaccine. However, these data on the 2-month residual immune memory after the second dose may help anticipate future needs to adapt the vaccination strategy among the older.

## Conclusion

Our results demonstrate that, with the recommended vaccination scheme (i.e., two doses of BNT162b2), both antibody and cellular responses are impaired in the COVID-19-naive older population compared with the younger group: this definitely confirms that specific studies are necessary to assess the immunogenicity of mRNA vaccines in frail older people (6, 7). Recent studies showed only a slightly lower immunogenicity after two doses with the delta variant that is currently responsible of a large majority of COVID-19 cases in many countries. We can suppose that the reduced immunogenicity of the BNT162b2 vaccine among older people may be similar or lower for the delta variant. For this reason, some countries have recently decided to recommend a third dose in older people and not only in immunocompromised patients. Our study illustrates that, even if the ability to respond to neoantigens is impaired in the older, the post-COVID-19 memory immune response is improved by an additional boost. Our work highlights the need of specific studies to assess the efficiency of SARS-CoV-2 mRNA-based vaccines in older people living in LCTFs, as in other immunocompromised subjects, and notably to confirm that a third dose may improve protective immunity.

## Data Availability Statement

The raw data supporting the conclusions of this article will be made available by the authors, without undue reservation.

## Ethics Statement

The studies involving human participants were reviewed and approved by the Ile-De-France V (ID‐CRB 2021-A00119-32) ethics committee. The patients/participants provided their written informed consent to participate in this study.

## Author Contributions

JD, BC-S, ML, and GL conceived and designed the study and participated in data collection, analysis, writing of the manuscript, and revision of the manuscript. EA, AG, JT, SM, FV, ArD, DH-G, JP, DaD, KF, DoD, LB, AlD, JL, AS, FP, and MH participated in data collection, analysis, and revision of the manuscript. All authors contributed to the article and approved the submitted version.

## Funding

This work was supported by the French government through the Programme Investissement d’Avenir (I-SITE ULNE/ANR-16-IDEX-0004 ULNE) managed by the Agence Nationale de la Recherche.

## Conflict of Interest

The authors declare that the research was conducted in the absence of any commercial or financial relationships that could be construed as a potential conflict of interest.

## Publisher’s Note

All claims expressed in this article are solely those of the authors and do not necessarily represent those of their affiliated organizations, or those of the publisher, the editors and the reviewers. Any product that may be evaluated in this article, or claim that may be made by its manufacturer, is not guaranteed or endorsed by the publisher.
