# Supplementary material for: Impaired Functional T-Cell Response to SARS-CoV-2 After Two Doses of BNT162b2 mRNA Vaccine in Older People
Source: Front Immunol. 2021 Nov 16;12:778679. doi: 10.3389/fimmu.2021.778679 (PMC8637126; doi:10.3389/fimmu.2021.778679)
Supplement: Supplementary file 1 [file DataSheet_1.pdf]

Supplementary Figures

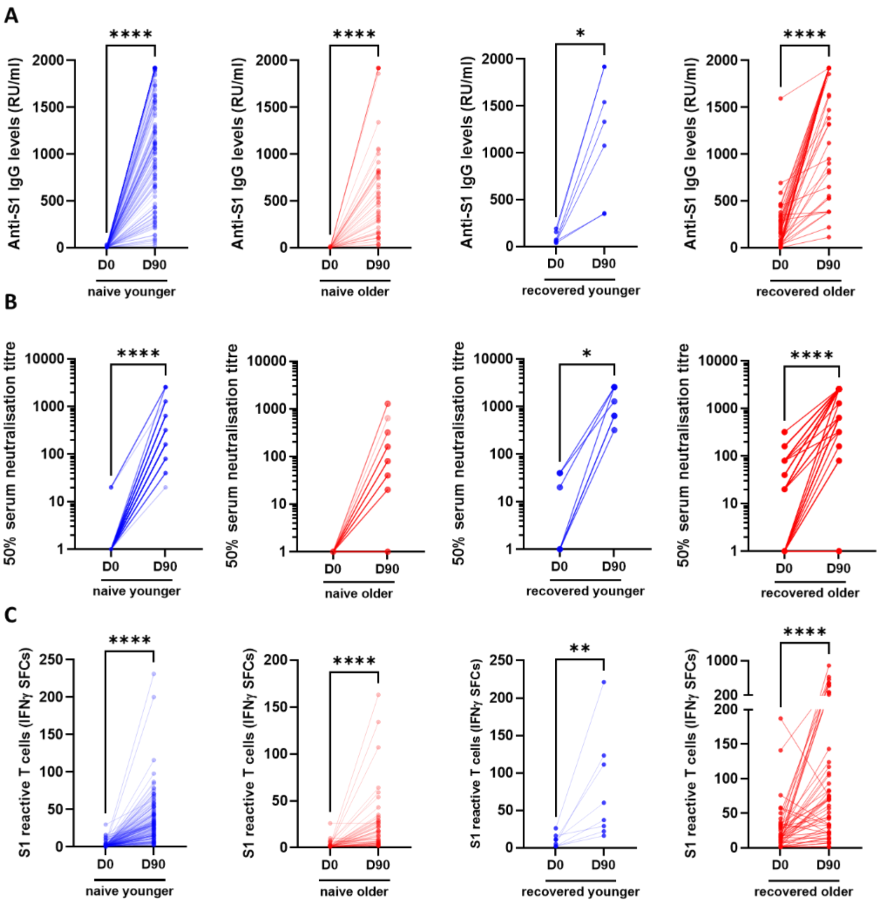

Supplementary Figure 1 | Specific antibody and T cell responses in older and in young adults before (D0) and 3 months (D90) after the first injection of BNT162b2. **(A)** Anti-S1 IgG, **(B)** serum neutralization assay against live virus and **(C)** S1 reactive T cells (ELISpot) in COVID-19-naïve and in COVID-19-recovered participants. Wilcoxon matched-pairs signed rank test was used for paired comparisons. P values * <0.05, ** <0.01, **** <0.0001. CTL, IFNγ SFCs, interferon gamma spot forming cells.

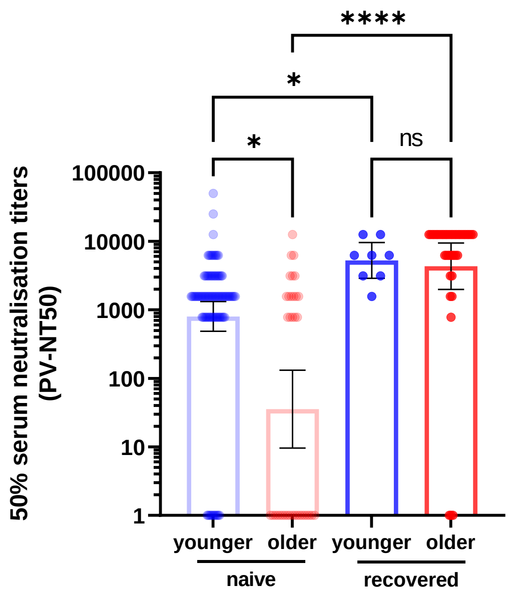

Supplementary Figure 2 | Serum neutralization assay against pseudovirus. This figure shows the 50% serum neutralization titer in a pseudovirus neutralization assay (PV-NT50) in COVID-19-naive younger adults (*n* = 103), COVID-19-naive older (*n* = 36), COVID-19 recovered younger adults (*n* = 8) and recovered older persons (*n* = 41). Although the method was different from the live virus assay, the results of neutralization from one group to another were similar. Geometric median and 95% confidence interval are shown. P values * < 0.05, ** < 0.01, *** < 0.001, **** < 0.0001, ns: not significant.

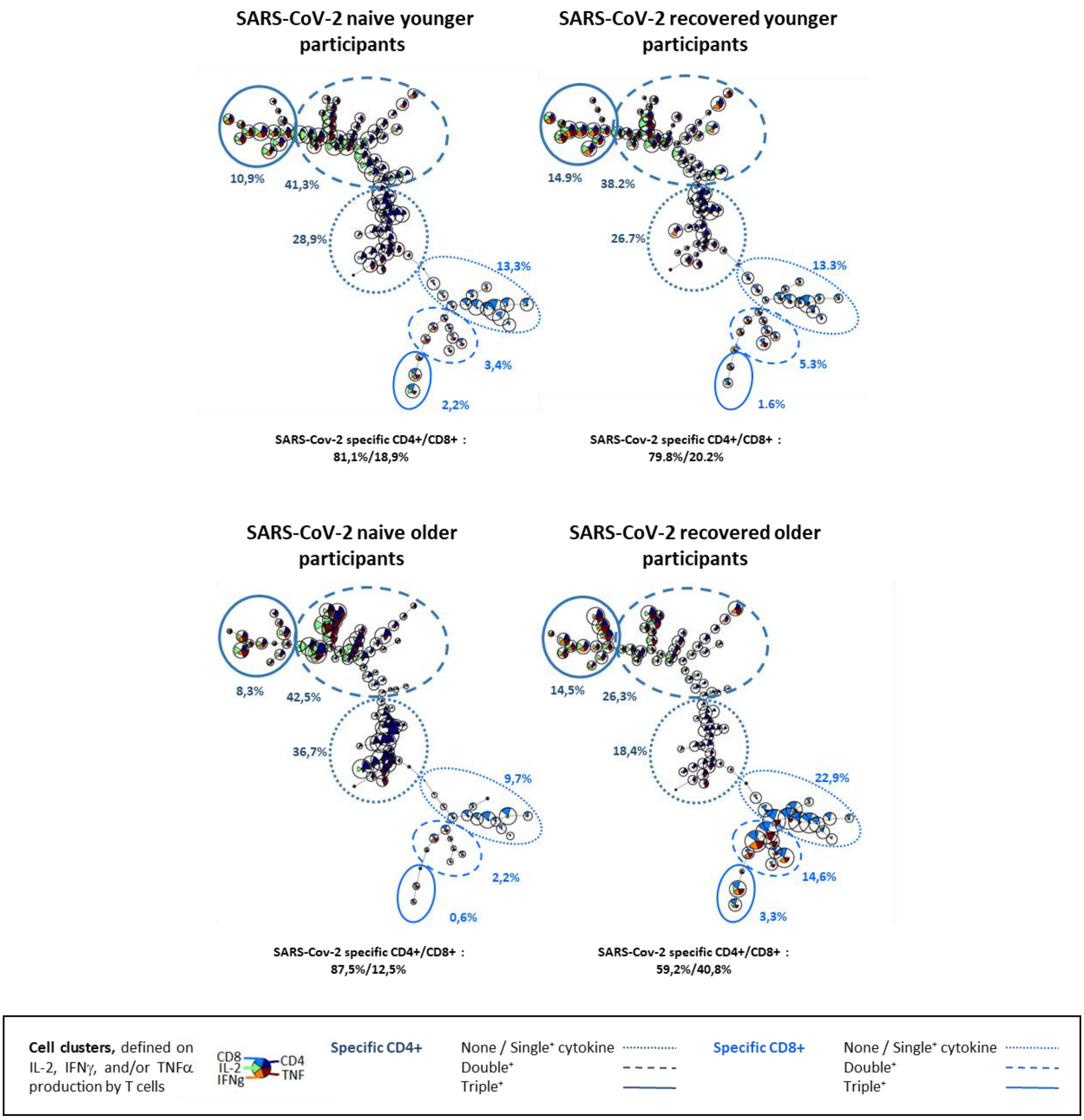

Supplementary Figure 3 | Cluster analysis of specific T cells subset after BNT162b2 in older and in young adults. FlowSOM results for COVID-19-naive and COVID-19-recovered young adults (top) and for COVID-19-naive and COVID-19-recovered older adults (bottom). Cell clusters were defined according to IL-2, IFNγ and TNFα expression. Manual metaclusters were identified among specific CD4+ T cells (dark blue) and specific CD8+ T cells (light blue) for cells producing none or one (small dotted line), two (large dotted line) or three cytokines (plain line) out of INFγ, IL-2 and TNFα.

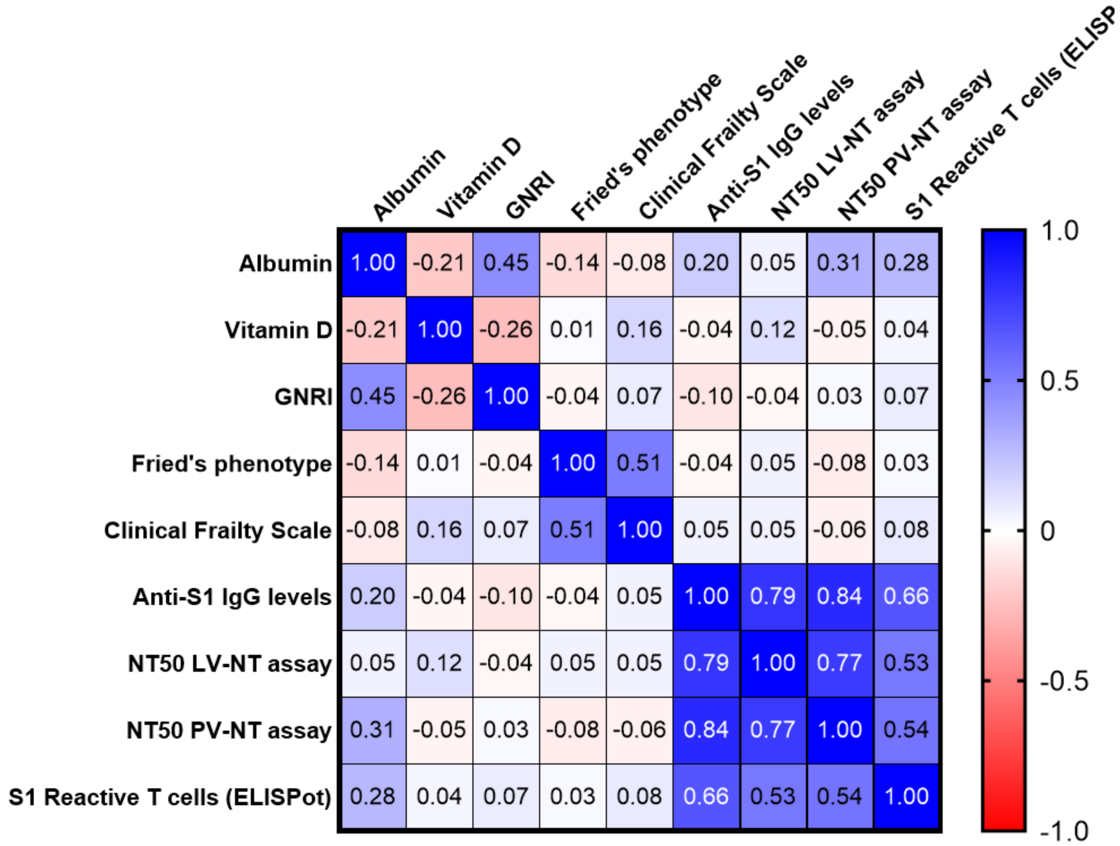

Supplementary Figure 4 | Correlations between nutritional status and frailty scale at baseline, and main immune parameters of the post vaccinal response at 3 months in COVID-19 naive older subjects. The values correspond to Spearman's rank correlation (r) coefficients. Only one correlation was found significant, between Albumin level and NT50 pV-NT (*r [95% CI)* 0.31 [0.022;0.55], *P* = 0.031, sample size *n* = 49). AIM^{+}, cell expressing activation induced markers; NT50 LV-NT assay, 50% serum neutralization titer in live virus neutralization assay; NT50 pV-NT assay, 50% serum neutralization titer in pseudovirus neutralization assay.

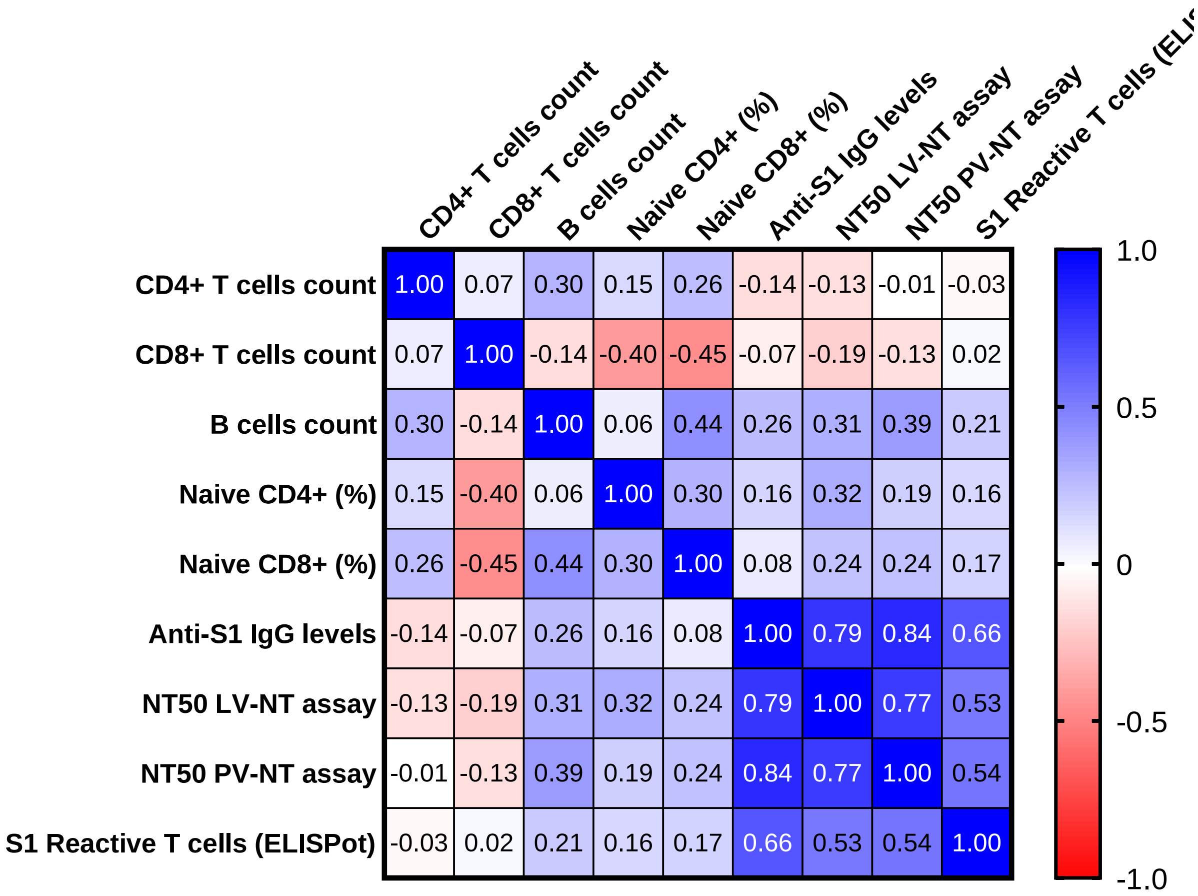

Supplementary Figure 5 | Correlations between T and B cells counts at baseline, and main immune parameters of the post vaccinal response at 3 months in COVID-19 naive older subjects. The values correspond to Spearman's rank correlation (r) coefficients. Only one correlation was found to be significant, between B cell count level and NT50 pV-NT (*r [95%CI)* 0.39 [0.009;0.63], $P = 0.012$, sample size $n = 41$). AIM$^+$, cell expressing activation induced markers; NT50 LV-NT assay, 50% serum neutralization titer in live virus neutralization assay; NT50 PV-NT assay, 50% serum neutralization titer in pseudovirus neutralization assay.
